# Dexamethasone dose reduction for suspected dexamethasone-related delirium during weekly paclitaxel/carboplatin-based concurrent chemoradiotherapy: a two-case report

**DOI:** 10.1186/s40780-026-00618-7

**Published:** 2026-08-14

**Authors:** Megumi Shingai, Karen T. Ishimatsu, Yuki Shigetsura, Natsuki Imayoshi, Noriko Kishi, Yasuaki Ikemi, Shunsaku Nakagawa, Tomohiro Terada

**Affiliations:** 1 Department of Clinical Pharmacology and Therapeutics, Kyoto University Hospital, 54 Shogoin- Kawahara-cho, Sakyo-ku, Kyoto, 606-8507 Japan; 2 Department of Radiation Oncology and Image-Applied Therapy, Graduate School of Medicine, Kyoto University, 54 Kawahara-cho, Shogoin, Sakyo- ku, Kyoto, 606-8507 Japan

**Keywords:** Weekly PAC/CBDCA, Radiotherapy, Reduction, Dexamethasone, Delirium

## Abstract

**Background:**

Dexamethasone (DEX), a type of corticosteroid, is widely used as an essential component of antiemetic prophylaxis in chemotherapy and chemoradiotherapy. However, corticosteroid is associated with adverse events, including delirium. Although dose reduction of the causative agent is generally recommended when adverse events occur, evidence regarding the feasibility of DEX dose reduction in emetogenic regimens, particularly weekly paclitaxel/carboplatin with concurrent radiotherapy (weekly PAC/CBDCA + RT), is limited.

**Case presentation:**

We present two cases of successful DEX dose reduction in patients undergoing weekly PAC/CBDCA + RT who developed delirium suspected to be associated with DEX. In both cases, DEX was initially administered at 6.6 mg for antiemetic prophylaxis. Following the development of delirium, the DEX dose was gradually decreased; the absence of nausea and vomiting was considered in assessing the feasibility of dose reduction. After dose reduction, the symptoms of delirium improved, with no apparent deterioration in antiemetic control based on objective clinical assessment, including CTCAE assessment and the absence of rescue antiemetic use. One patient discontinued chemotherapy because of persistent hematologic toxicity.

**Conclusions:**

These two cases suggest that a gradual reduction of DEX dosage may be considered as a potential management strategy for selected patients undergoing weekly PAC/CBDCA + RT when steroid-related adverse events, such as delirium, occur. Careful monitoring and personalized adjustment of antiemetic regimens may facilitate the treatment continuation while maintaining antiemetic control of treatment, even in moderate emetogenic chemoradiotherapy settings.

## Background

Concurrent chemotherapy and radiotherapy represent the established standard of care for patients with unresectable, locally advanced stage III non-small cell lung cancer (NSCLC). In Japan, a regimen of weekly paclitaxel/carboplatin therapy with radiotherapy (weekly PAC/CBDCA + RT) is frequently employed for NSCLC [1] Antiemetic prophylaxis for chemotherapy-induced nausea and vomiting (CINV) is guided by established guidelines based on the emetogenicity of the agents [2, 3]. As both chemotherapy and radiotherapy contribute to emetic symptoms [4], weekly PAC/CBDCA + RT carries substantial emetogenic potential. Accordingly, antiemetic prophylaxis typically combines a 5-HT_3_ receptor antagonist and dexamethasone (DEX), based on current guideline recommendations [3].

DEX is an essential component of antiemetic prophylaxis in weekly PAC/CBDCA + RT. Notably, it is the only corticosteroid approved for the treatment of CINV, with demonstrated significant efficacy both as a monotherapy and in combination with other antiemetic agents. Furthermore, DEX is recognized by the American Society of Clinical Oncology guidelines as an effective agent for managing radiotherapy-induced nausea and vomiting (RINV) [2]. However, observational studies involving patients with cancer demonstrated that corticosteroid use is associated with various adverse effects, including neuropsychiatric symptoms such as delirium [5, 6]. In clinical practice, corticosteroid-associated adverse events may necessitate dose reduction or discontinuation of the causative agent [7]. While such scenarios are relatively uncommon during weekly PAC/CBDCA + RT, they are occasionally encountered. Despite this clinical need, no evidence exists on whether DEX can be safely reduced in this regimen, which carries a substantial emetogenic risk and in which DEX is considered indispensable for antiemetic prophylaxis.

In this report, we present two cases of patients undergoing weekly PAC/CBDCA + RT who developed delirium suspected to be associated with DEX, in whom dose reduction was successfully implemented. In both cases, stepwise DEX dose reduction was achieved without compromising antiemetic control or treatment completion. Although limited in number, these cases provide practical clinical insights into DEX dose adjustment when steroid-related adverse events necessitate intervention, an area with limited existing guidance.

## Case presentation

### Case 1

The first case was a male patient in his 70s (performance status 0) with a history of hypertension, dyslipidemia, postoperative colon cancer, and no known allergies. He had smoked approximately 20 cigarettes per day since his 20s, but had quit smoking a few months prior to presentation owing to a persistent cough. In April, he was referred to Kyoto University Hospital, where positron emission tomography-computed tomography (PET-CT) and cytological examination led to a diagnosis of right upper lobe lung adenocarcinoma, cT1bN2M0, Stage IIIA. Consequently, the patient was admitted for radical chemotherapy. His outpatient medications included carbocisteine (1500 mg/day), tipepidine hibenzate (60 mg/day), clenbuterol (80 mg/day), sulpiride (100 mg/day), and metoclopramide (15 mg/day).

**Fig. 1:**
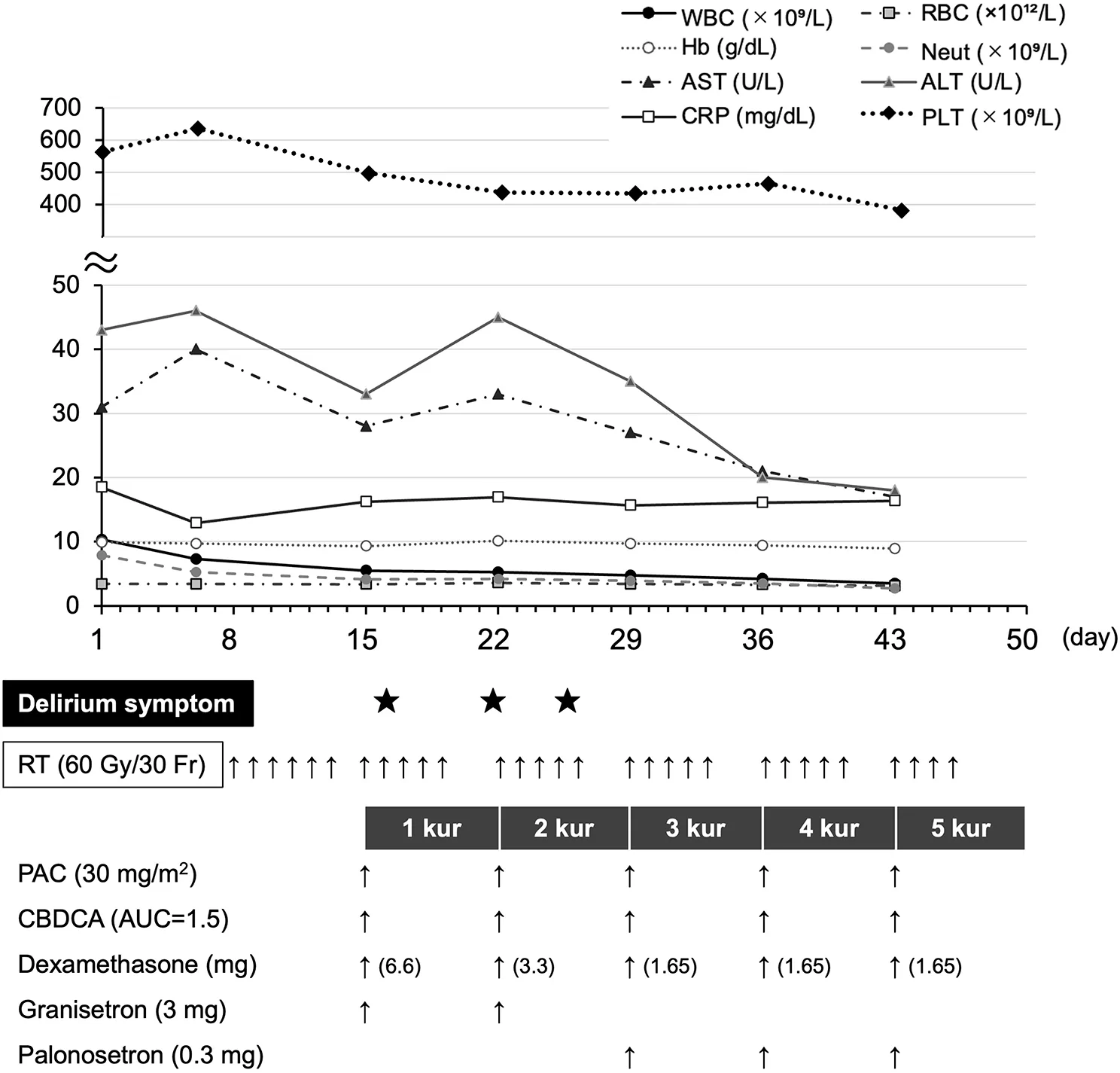
Clinical course of Case 1. Progression of laboratory values, chemotherapy regimen schedule, and timing of delirium onset for Case 1 throughout the course of treatment. RT: radiotherapy, PAC: paclitaxel, CBDCA: carboplatin, WBC: white blood cell, RBC: red blood cell, Neut: neutrophil, PLT: platelet, Hb: hemoglobin, AST: aspartate aminotransferase, CRP: C-reactive protein, ALT: alanine aminotransferase; AUC, area under the curve

**Fig. 2:**
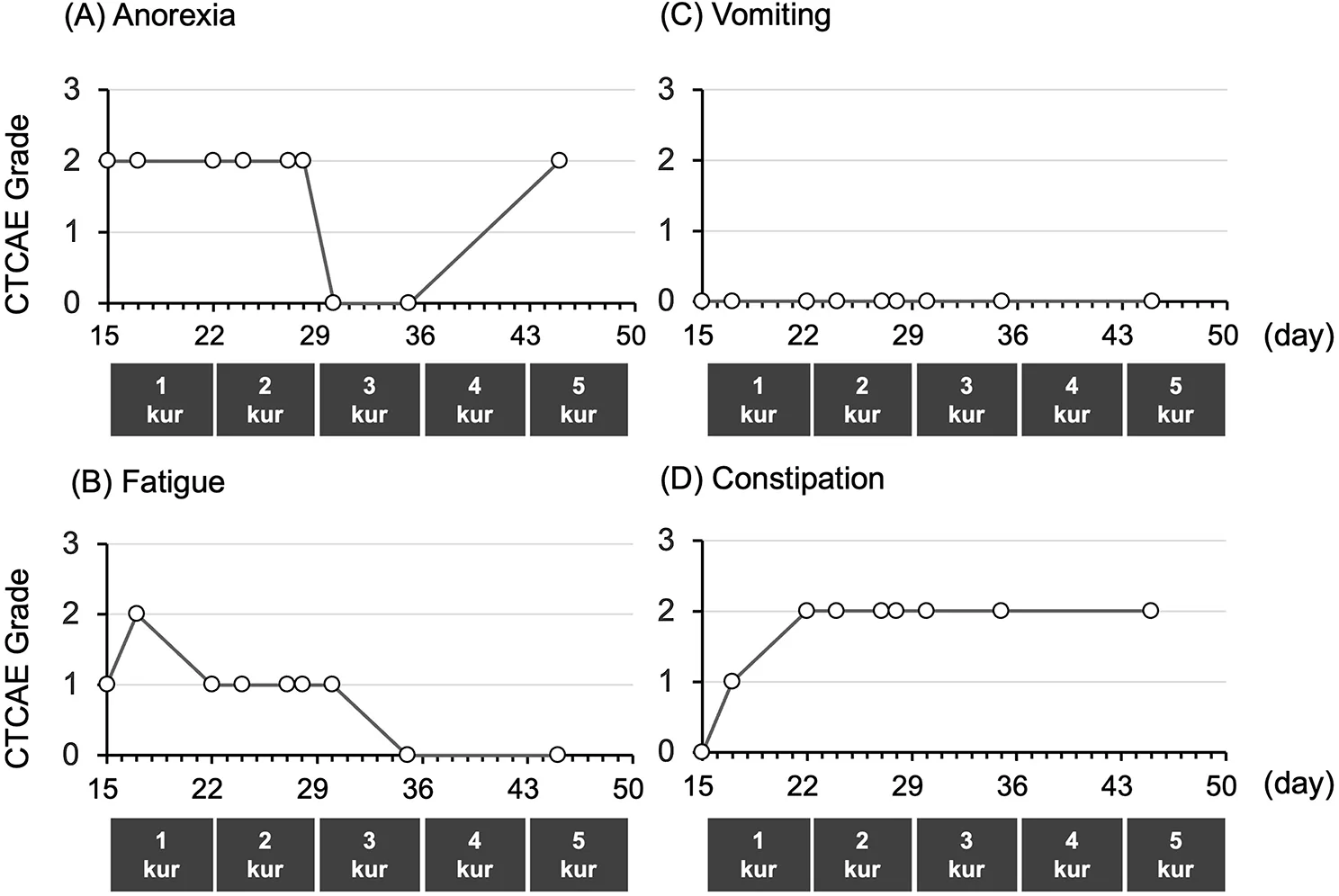
Changes in the CTCAE Grade for Case 1. The CTCAE grade progression of adverse events observed throughout the course of weekly PAC/CBDCA therapy and DEX dose adjustments is presented. (**A**) Anorexia, (**B**) Fatigue, (**C**) Vomiting, (**D**) Constipation. The CTCAE grades shown were prospectively assessed during routine clinical care and documented in the electronic medical records. CTCAE: common terminology criteria for adverse events

The clinical course is presented in Fig. 1. Adverse events were graded using the common terminology criteria for adverse events (CTCAE) version 5.0 (Fig. 2). PAC and CBDCA were initially scheduled for administration on day 2 but were postponed owing to elevated white blood cell (WBC) count and C-reactive protein (CRP). On day 7, the blood culture results were negative, and the antimicrobial therapy was switched to ampicillin-sulbactam (3 g every 6 h). Consequently, chemotherapy was delayed, and radiotherapy alone (60 Gy/30 Fr) was initiated seven days before chemotherapy.

By day 14, the signs of infection had resolved, antimicrobials were discontinued, and the WBC count had normalized. Weekly PAC/CBDCA was initiated on day 15. However, PAC (30 mg/m^2^) and CBDCA (area under the curve: AUC 1.5) were administered at doses lower than those in the institutional standard regimen (Table 1). Antiemetic prophylaxis consisted of DEX (6.6 mg) and granisetron (3 mg).

**Table 1:** Overview of the weekly paclitaxel/carboplatin therapy regimen at Kyoto University Hospital

Agent	Day 1	Day 2	Day 3	Day 4	Day 5
Paclitaxel (40 mg/m^2^)	○				
Carboplatin (area under the curve: 2)	○				
Radiotherapy (60 Gy/30 Fr)	○	○	○	○	○
Dexamethasone 6.6 mg	○				
Granisetron 3 mg	○				
*d*-Chlorpheniramine Maleate 5 mg	○				
Famotidine 20 mg	○				

On day 16, the patient developed nocturnal restlessness, wandering, hallucinations, and delusions. Based on the acute onset and fluctuating course of these neuropsychiatric symptoms, including impaired attention and orientation, the attending physicians clinically suspected delirium and managed the patient accordingly. Although a validated delirium assessment tool was not used during the clinical course, retrospective assessment using the Confusion Assessment Method (CAM) [8] based on the physicians’ and nurses’ medical records demonstrated the presence of (1) acute onset and fluctuating course, (2) inattention, (3) disorganized thinking, and (4) altered level of consciousness, fulfilling the CAM diagnostic criteria for delirium. Advanced age, recent infection, hospitalization, advanced cancer, concurrent radiotherapy, chemotherapy, and concomitant medications were considered as potential contributing factors [9–12]. However, the infection had clinically resolved, antimicrobial therapy had been discontinued, and inflammatory markers had improved before symptom onset. Furthermore, concomitant dopamine antagonists had been administered at stable doses before admission without neuropsychiatric symptoms. Considering the temporal relationship between DEX administration and symptom onset, DEX-associated delirium was suspected. Although risperidone 0.5 mg was prescribed as needed, he declined it owing to poor insight. Delirium symptoms partially improved the next day but recurred as nocturnal restlessness and insomnia on day 21. These findings were deemed consistent with corticosteroid-related delirium based on the acute onset of neuropsychiatric symptoms in temporal association with DEX, as assessed by the physicians. Accordingly, the DEX dose was reduced to 3.3 mg for subsequent cycles, considering the absence of chemotherapy-induced nausea and vomiting after the initial cycle. On day 22, DEX (3.3 mg) was administered in combination with PAC (30 mg/m^2^), CBDCA (AUC 1.5), and granisetron (3 mg).

On day 23, fatigue (CTCAE grade 2) and dysphagia were observed, and the dosages of PAC/CBDCA were accordingly adjusted. On day 24, nocturnal hallucinations, delusions, and insomnia recurred. In response to the recurrence of delirium, the DEX dose was further reduced to 1.65 mg on day 29. In addition, granisetron was replaced with palonosetron, as it possesses stronger antiemetic efficacy [13], and palonosetron (0.3 mg) was administered at a low dose to manage concurrent constipation.

Subsequently, the patient experienced no further episodes of delirium. Vomiting remained at CTCAE Grade 0 throughout the subsequent treatment cycles, and no rescue antiemetic medication was required. Anorexia was assessed as CTCAE Grade 2 from Day 15 to Day 28, while oral intake gradually decreased from approximately 100% at the start of treatment to approximately 30% after Day 8, and body weight decreased from 57.6 kg before treatment to 52.8 kg by Day 25. No clinical symptoms suggestive of CINV were documented. Because vomiting remained absent, these changes were considered likely to be multifactorial, reflecting the underlying lung cancer, persistent cough, fatigue, and the overall effects of concurrent chemoradiotherapy rather than inadequate control of chemotherapy-induced nausea and vomiting. Chemotherapy was administered on days 36 and 43 using the same regimen, and treatment was completed without further complications.

## Case 2

The second case involved a female patient in her 70s (performance status 0) with a medical history of osteoporosis, with no known drug or food allergies, and no history of asthma. Her smoking history consisted of 10 cigarettes per day from age 25 for 20 years, followed by 2–3 cigarettes per day until she quit one year prior to admission. She reported occasional alcohol consumption, limited to one glass at a time.

Approximately one month before admission, she developed a cough and underwent chest CT at her primary care clinic, which revealed a suspected right lower lobe lung cancer, cT2aN1M0, STAGE IIB, with ipsilateral hilar and mediastinal lymph node metastases. Based on the clinical assessment, concurrent chemoradiotherapy was recommended, and the patient was admitted for treatment. Her outpatient medications included flusultiamine (50 mg/day) and sennoside (12 mg/day), as well as supplements such as coenzyme Q10 and iron/folic acid.

**Fig. 3:**
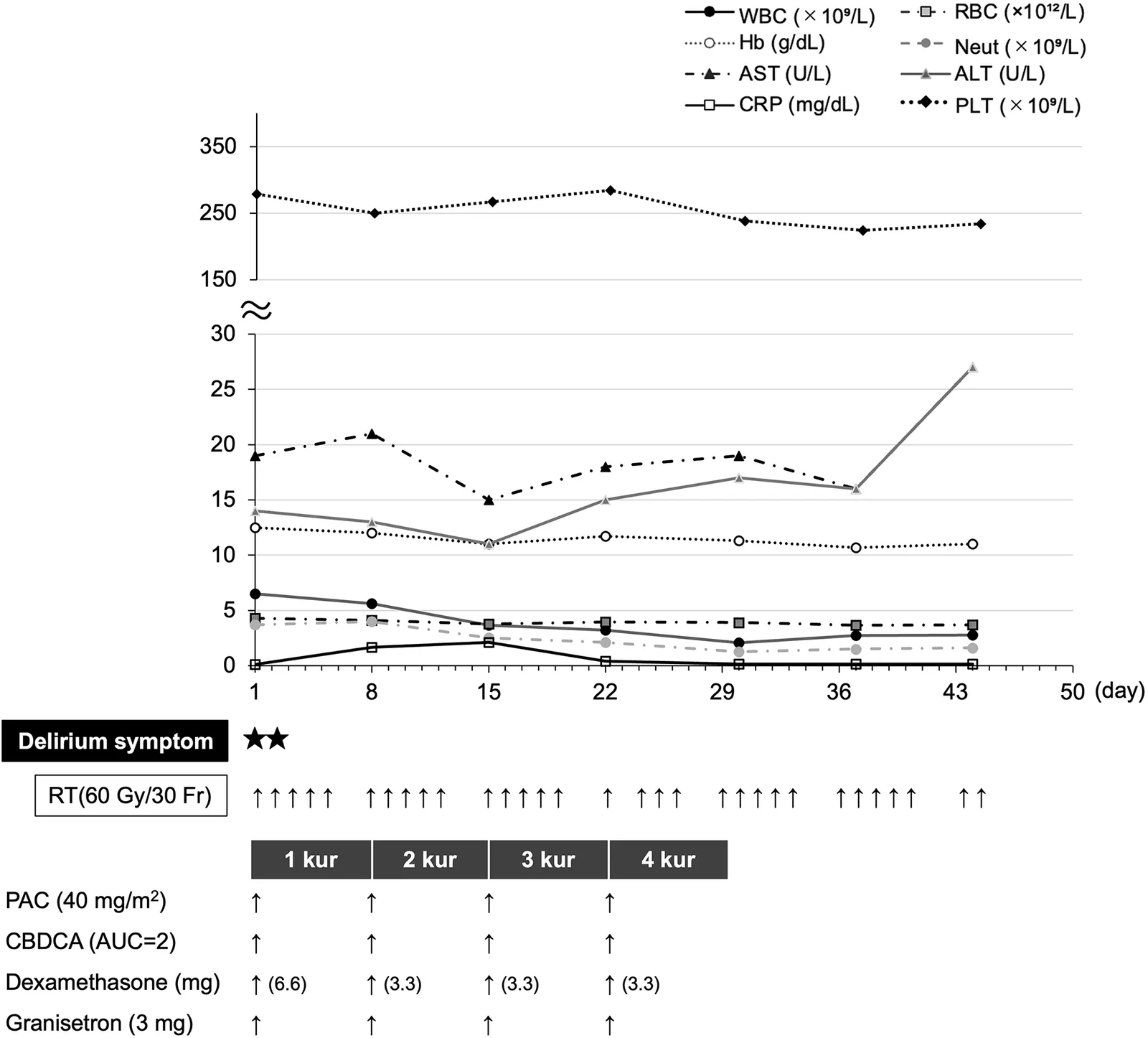
Clinical course of Case 2. Progression of laboratory values, chemotherapy regimen schedule, and timing of delirium onset for Case 2 throughout the course of treatment are shown. RT: radiotherapy, PAC: paclitaxel, CBDCA: carboplatin, WBC: white blood cell, RBC: red blood cell, Neut: neutrophil, PLT: platelet, Hb: hemoglobin, AST: aspartate aminotransferase, CRP: C-reactive protein, ALT: alanine aminotransferase; AUC, area under the curve

**Fig. 4:**
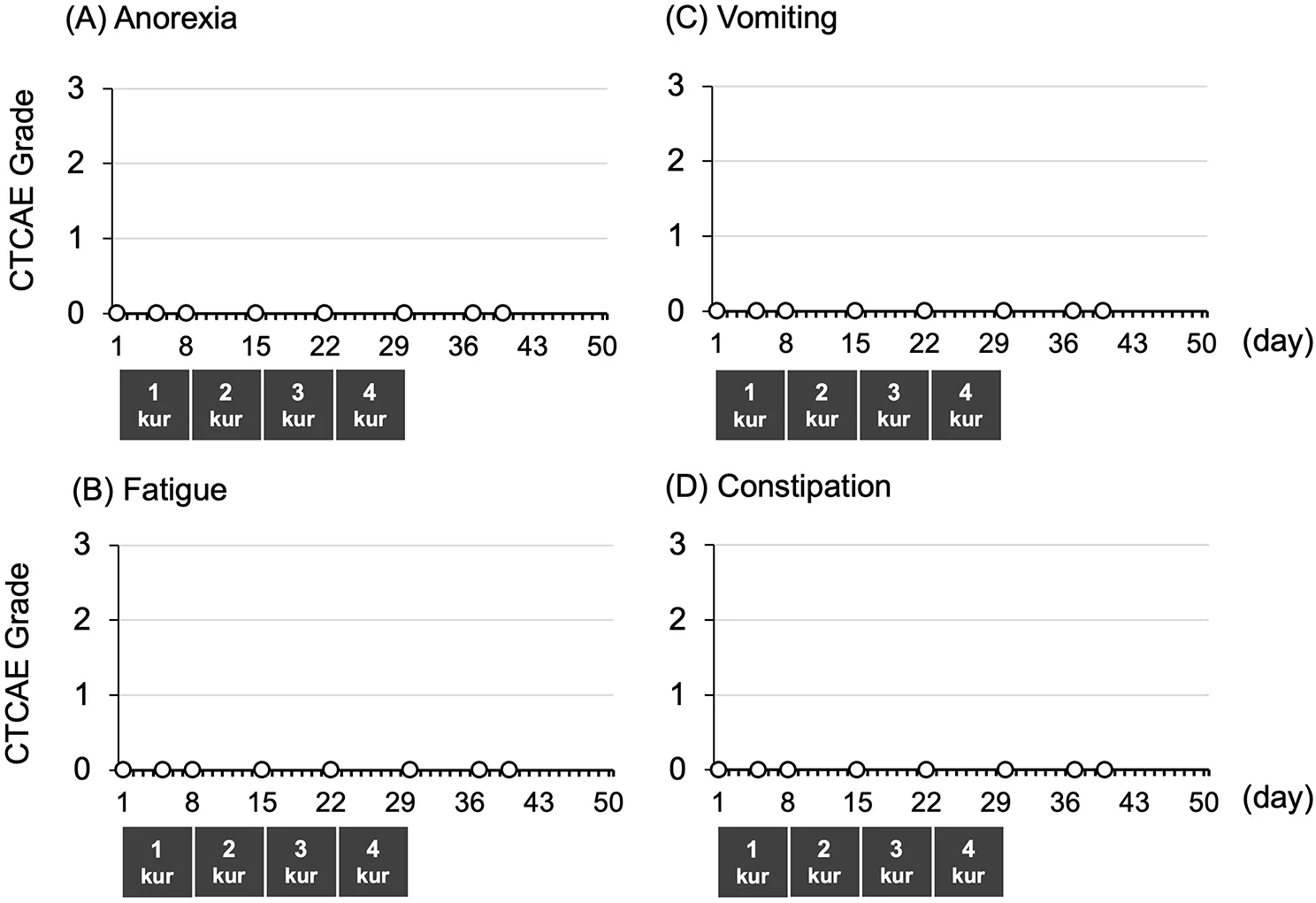
Changes in the CTCAE Grade for Case 2. The CTCAE grade progression of adverse events observed throughout the course of weekly PAC/CBDCA therapy and DEX dose adjustments is presented. (**A**) Anorexia, (**B**) Fatigue, (**C**) Vomiting, (**D**) Constipation. The CTCAE grades shown were prospectively assessed during routine clinical care and documented in the electronic medical records. CTCAE: common terminology criteria for adverse events

The clinical course is presented in Fig. 3, and adverse events were graded according to the CTCAE version 5.0 (Fig. 4). Chemotherapy was initiated according to our hospital’s protocol concurrently with radiotherapy on day 1 (Table 1). No allergic reactions, nausea, vomiting, or appetite loss were observed during or immediately after the first infusion (day 1). However, after the first infusion, the patient exhibited confusion, including forgetting that the infusion had ended, looking anxiously out of the room, and showing temporal disorientation. On the morning of day 2, she questioned the purpose of her indwelling needle and exhibited excessive concern regarding items near the room entrance. Based on the acute onset and fluctuating course of these neuropsychiatric symptoms, the attending physicians clinically suspected delirium and managed the patient accordingly. Retrospective assessment using the CAM [8] based on the physicians’ and nurses’ medical records demonstrated the presence of (1) acute onset and fluctuating course, (2) inattention, and (3) disorganized thinking, fulfilling the CAM diagnostic criteria for delirium. Potential contributing factors, including advanced age, hospitalization, malignancy, concurrent chemoradiotherapy, chemotherapy, and concomitant medications, were considered [9–12]. No apparent acute medical conditions that could better explain the symptoms were identified. Considering the temporal relationship between DEX administration and symptom onset, DEX-associated delirium was suspected. Accordingly, the DEX dose was reduced from 6.6 mg to 3.3 mg for the second infusion on day 8, with careful monitoring for nausea and fatigue.

Between the second and third infusions, the patient exhibited no further symptoms of delirium. Therefore, DEX 3.3 mg was continued for the third infusion on day 16. Throughout the subsequent treatment cycles, vomiting and anorexia remained at CTCAE Grade 0, and no rescue antiemetic medication was required. Oral intake remained approximately 100% throughout treatment, and body weight remained relatively stable, changing from 46.5 kg at baseline to 45.1 kg on Day 18. No patient-reported symptoms suggestive of CINV were documented after dexamethasone dose reduction. Accordingly, the same DEX dose was maintained for the fourth infusion (Day 24).

No recurrence of delirium was observed after the DEX dose reduction. No increase in CTCAE grade of nausea, vomiting, or appetite loss was observed, and oral intake was maintained without clinically significant deterioration. However, before the next scheduled infusion, chemotherapy was postponed owing to Grade 2 leukopenia and Grade 2 neutropenia. As these hematologic toxicities persisted at Grade 2 the following week, chemotherapy was discontinued, while radiotherapy alone was continued and completed without complications.

## Discussion and conclusions

In chemotherapy regimens containing CBDCA, the combination of a 5-HT_3_ receptor antagonist and DEX is recommended for antiemetic prophylaxis [2, 3] DEX is crucial for preventing CINV and RINV and is generally considered an essential component of weekly PAC/CBDCA + RT. However, DEX is associated with various adverse effects, including delirium. Delirium is common among hospitalized patients with advanced cancer, and observational studies have identified corticosteroid exposure as an independent risk factor for delirium, particularly at higher corticosteroid doses [14, 15]. When delirium occurs, dose reduction or discontinuation of DEX is generally recommended in clinical practice [7]. A key clinical challenge is whether the dose of DEX can be safely reduced in weekly PAC/CBDCA + RT. However, there is currently no evidence regarding the feasibility of DEX dose reduction in this specific treatment setting. Reducing DEX in weekly PAC/CBDCA + RT raises concerns about compromising antiemetic efficacy. Although evidence on this approach remains limited, several reports have described stepwise dose reduction of DEX in other chemotherapy regimens involving PAC or CBDCA [16]. In these reports, DEX was reduced to one-half to one-third of the initial dose to mitigate steroid-related adverse effects without affecting treatment feasibility. However, these reports are not specific to concurrent chemoradiotherapy. In the present report, DEX dose reduction was performed using a similar stepwise approach. This strategy was informed by previous reports suggesting that tapering DEX after confirming the absence of PAC-related hypersensitivity reactions may reduce cumulative corticosteroid exposure without increasing the risk of hypersensitivity reactions [17]. Unlike those reports, which evaluated planned DEX tapering in patients receiving weekly paclitaxel for advanced non-small cell lung cancer, the present cases involved individualized DEX dose reduction from 6.6 mg to 3.3 mg or 1.65 mg in patients undergoing concurrent chemoradiotherapy because of suspected DEX-related delirium. Another study also demonstrated that DEX could be safely discontinued after the first paclitaxel administration in patients without hypersensitivity reactions [18, 19].

In both cases, based on objective clinical findings, including CTCAE grading and the absence of rescue antiemetic use, no clinically significant worsening of nausea, vomiting, or hypersensitivity reactions was observed, even after reducing the dose to 3.3 mg or 1.65 mg. This may be partly explained by the concomitant use of 5-HT_3_ receptor antagonists and, in one case, switching to palonosetron, which has a longer half-life and stronger antiemetic efficacy than granisetron [20, 21]. In addition, the relatively low dose of CBDCA (AUC: 1.5) and the weekly administration schedule may have contributed to maintaining adequate antiemetic control despite DEX reduction. In the present cases, no hypersensitivity reactions occurred after reducing DEX to 1.65 mg. However, these findings are insufficient to conclude that this dose is adequate for hypersensitivity prophylaxis. Further studies are needed to determine the minimum effective DEX dose for preventing paclitaxel-associated hypersensitivity reactions.

Although several established risk factors for delirium were present in both patients, including advanced age, hospitalization, concurrent chemoradiotherapy, and concomitant medications (including dopamine antagonists in Case 1), the temporal relationship between DEX administration, dose reduction, and symptom resolution was considered consistent with DEX being the most plausible precipitating factor. In Case 1, delirium persisted after the DEX dose was reduced from 6.6 mg to 3.3 mg and resolved only after a further reduction to 1.65 mg. Therefore, the improvement cannot be attributed simply to the initial dose reduction. Rather, the clinical course suggests that symptom resolution was temporally associated with stepwise DEX dose reduction. Previous studies [22] have suggested a dose-dependent association between corticosteroid exposure and neuropsychiatric adverse effects, including delirium. Accordingly, it is possible that the dose of 3.3 mg remained above this patient’s individual susceptibility threshold, whereas further reduction to 1.65 mg lowered DEX exposure sufficiently to permit symptom resolution. However, this interpretation remains speculative and cannot establish a causal or dose-response relationship.

Overall, these findings suggest that, with appropriate monitoring and supportive care, DEX dose reduction may be feasible in selected patients undergoing weekly PAC/CBDCA + RT.

Weekly PAC/CBDCA + RT is widely used for the treatment of locally advanced NSCLC [23], and cases requiring DEX dose reduction may be encountered in routine clinical practice. However, evidence to guide such adjustments remains lacking. Despite the limited number of cases, the present report provides clinically meaningful insights into a potential management approach for DEX dose reduction in this commonly used, moderate emetogenic chemoradiotherapy regimen.

In conclusion, these cases highlight a possible clinical approach in which stepwise DEX dose reduction may be considered in selected patients undergoing weekly PAC/CBDCA + RT who experience steroid-related adverse events. In these two cases, DEX dose reduction was associated with improvement in delirium without apparent deterioration in antiemetic control under careful monitoring and individualized adjustment of antiemetic therapy. Given the limited number of cases, these findings should be regarded as hypothesis-generating. Further studies are warranted to establish optimal strategies for corticosteroid dose adjustment in emetogenic therapy.

## Data Availability

No datasets were generated or analysed during the current study.

## Abbreviations

AUC: Area under the curve
CAM: Confusion Assessment Method
CBDCA: Carboplatin
CINV: Chemotherapy-induced nausea and vomiting
CRP: C-reactive protein
CTCAE: Common Terminology Criteria for Adverse Events
DEX: Dexamethasone
HSR: Hypersensitivity reactions
PAC: Paclitaxel
RINV: Radiotherapy-induced nausea and vomiting
WBC: White blood cell

## References

[1] Yamamoto N, Nakagawa K, Nishimura Y, Tsujino K, Satouchi M, Kudo S, et al. Phase III study comparing second- and third-generation regimens with concurrent thoracic radiotherapy in patients with unresectable stage III non-small-cell lung cancer: West Japan Thoracic Oncology Group WJTOG0105. J Clin Oncol. 2010;28:3739–45. 10.1200/JCO.2009.24.5050. 20625120

[2] Hesketh PJ, Kris MG, Basch E, Bohlke K, Barbour SY, Clark-Snow RA, et al. Antiemetics: American Society of Clinical Oncology Clinical Practice Guideline Update. J Clin Oncol. 2017;35:3240–61. 10.1200/JCO.2017.74.4789. 28759346

[3] Hesketh PJ, Kris MG, Basch E, Bohlke K, Barbour SY, Clark-Snow RA, et al. Antiemetics: ASCO guideline update. J Clin Oncol. 2020;38:2782-97. 10.1200/JCO.20.01296. Erratum in: J Clin Oncol. 2020;38:3825. doi: 10.1200/JCO.20.02904. Erratum in: J Clin Oncol. 2021;39:96. doi: 10.1200/JCO.20.03378. PMID: 32658626. 32658626

[4] Feyer P, Jahn F, Jordan K. Radiation induced nausea and vomiting. Eur J Pharmacol. 2014;722:165–71. 10.1016/j.ejphar.2013.09.069. 24157983

[5] Gaudreau JD, Gagnon P, Harel F, Roy MA, Tremblay A. Psychoactive medications and risk of delirium in hospitalized cancer patients. J Clin Oncol. 2005;23:6712–8. 10.1200/JCO.2005.05.140. 16170179

[6] Uchida M, Okuyama T, Ito Y, Nakaguchi T, Miyazaki M, Sakamoto M, et al. Prevalence, course and factors associated with delirium in elderly patients with advanced cancer: a longitudinal observational study. Jpn J Clin Oncol. 2015;45:934–40. 10.1093/jjco/hyv100. 26185141

[7] Judd LL, Schettler PJ, Brown ES, Wolkowitz OM, Sternberg EM, Bender BG, et al. Adverse consequences of glucocorticoid medication: psychological, cognitive, and behavioral effects. Am J Psychiatry. 2014;171:1045–51. 10.1176/appi.ajp.2014.13091264. Erratum in: in Am J Psychiatry. 2014;171:1224. doi: 10.1176/appi.ajp.2014.17111correction. 25272344

[8] Watanabe A. The Confusion Assessment Method: Validation of Japanese-language version. Jpn J Gen Psychiatry. 2013;25:165–70. 10.11258/jjghp.25.165.

[9] Ahmed S, Leurent B, Sampson EL. Risk factors for incident delirium among older people in acute hospital medical units: a systematic review and meta-analysis. Age Ageing. 2014;43:326–33. 10.1093/ageing/afu022. PMC4001175 24610863

[10] Aldemir M, Ozen S, Kara IH, Sir A, Baç B. Predisposing factors for delirium in the surgical intensive care unit. Crit Care. 2001;5:265–70. 10.1186/cc1044. PMC83853 11737901

[11] Siddiqi N, House AO, Holmes JD. Occurrence and outcome of delirium in medical in-patients: a systematic literature review. Age Ageing. 2006;35:350–64. 10.1093/ageing/afl005. 16648149

[12] Jung P, Puts M, Frankel N, Syed AT, Alam Z, Yeung L, et al. Delirium incidence, risk factors, and treatments in older adults receiving chemotherapy: A systematic review and meta-analysis. J Geriatr Oncol. 2021;12:352–60. 10.1016/j.jgo.2020.08.011. 32943360

[13] Saito M, Aogi K, Sekine I, Yoshizawa H, Yanagita Y, Sakai H, et al. Palonosetron plus dexamethasone versus granisetron plus dexamethasone for prevention of nausea and vomiting during chemotherapy: a double-blind, double-dummy, randomised, comparative phase III trial. Lancet Oncol. 2009;10:115–24. 10.1016/S1470-2045(08)70313-9. published correction appears in Lancet Oncol. 2010;11(3)226. 19135415

[14] Uchida M, Okuyama T, Ito Y, Nakaguchi T, Miyazaki M, Sakamoto M, et al. Prevalence, course and factors associated with delirium in elderly patients with advanced cancer: a longitudinal observational study. Jpn J Clin Oncol. 2015;45:934–40. 10.1093/jjco/hyv100. 26185141

[15] Gaudreau JD, Gagnon P, Harel F, Roy MA, Tremblay A. Psychoactive medications and risk of delirium in hospitalized cancer patients. J Clin Oncol. 2005;23:6712–8. 10.1200/JCO.2005.05. 16170179

[16] Yoshizawa Y, Goto T, Matsubara T, Maezawa K, Mizukami H, Saito M, et al. A case of effective weekly paclitaxel administration for advanced gastric cancer associated with diabetes mellitus -about the control of blood glucose when dexamethasone for prophylaxis against paclitaxel-associated hypersensitivity reactions to the diabetic has been administered. Gan Kagaku Ryoho. 2008;35:641–44. PMID: 18408435. 18408435

[17] Nokihara H, Yamamoto N, Ohe Y, Hiraoka M, Tamura T. Pharmacokinetics of weekly paclitaxel and feasibility of dexamethasone taper in Japanese patients with advanced non-small cell lung cancer. Clin Ther. 2016;38:338–47. 10.1016/j.clinthera.2015.12.009. 26774841

[18] Quock J, Dea G, Tanaka M, Gandara D, Lara P, Lau D. Premedication strategy for weekly paclitaxel. Cancer Invest. 2002;20:666–72. 10.1081/cnv-120003535. 12197222

[19] Braverman AS, Rao S, Salvatti ME, Adamson B, McManus M, Pierre S. Tapering and discontinuation of glucocorticoid prophylaxis during prolonged weekly to biweekly paclitaxel administration. Chemotherapy. 2005;51:116–9. 10.1159/000085618. 15886470

[20] Rojas C, Stathis M, Thomas AG, et al. Palonosetron exhibits unique molecular interactions with the 5-HT3 receptor. Anesth Analg. 2008;107(2):469–78. 10.1213/ane.0b013e318172fa74. 18633025

[21] Rojas C, Thomas AG, Alt J, et al. Palonosetron triggers 5-HT(3) receptor internalization and causes prolonged inhibition of receptor function. Eur J Pharmacol. 2010;626(2–3):193–9. 10.1016/j.ejphar.2009.10.002. 19836386

[22] Wu Z, Li H, Liao K, Wang Y. Association Between Dexamethasone and Delirium in Critically Ill Patients: A Retrospective Cohort Study of a Large Clinical Database. J Surg Res. 2021;263:89–101. 10.1016/j.jss.2021.01.027. 33639374

[23] Bradley JD, Paulus R, Komaki R, et al. Standard-dose versus high-dose conformal radiotherapy with concurrent and consolidation carboplatin plus paclitaxel with or without cetuximab for patients with stage IIIA or IIIB non-small-cell lung cancer (RTOG 0617): a randomised, two-by-two factorial phase 3 study. Lancet Oncol. 2015;16(2):187–99. 10.1016/S1470-2045(14)71207-0. PMC4419359 25601342

